# Development of selective isolation media for detecting the genera Actinomyces and Schaalia from oral specimens containing indigenous bacteria

**DOI:** 10.1099/acmi.0.000768.v3

**Published:** 2024-05-10

**Authors:** Sadao Aoki, Hiroko Yahara, Ryoma Nakao, Osamu Tsuzukibashi, Koji Yahara

**Affiliations:** 1 Antimicrobial Resistance Research Center, National Institute of Infectious Diseases, Tokyo, Japan; 2 Genome Medical Science Project, Research Institute, National Center for Global Health and Medicine, Tokyo, Japan; 3 Department of Bacteriology I, National Institute of Infectious Diseases, Tokyo, Japan; 4 Department of Laboratory Medicine for Dentistry for the Compromised Patient, Nihon University School of Dentistry at Matsudo, Chiba, Japan

**Keywords:** *Actinomyces*, isolation, oral, selective media, swab

## Abstract

To isolate specific bacteria from samples constituting the microbiota, it is essential to employ selective media that suppress the growth of resident bacteria other than specific target bacteria. Selective media for clinically important *Actinomyces* (including *Schaalia*, which was previously taxonomically classified as part of the genus *Actinomyces*) have been limited because they have been designed for a limited range of species within the genus and require ingredients which are difficult to prepare and handle. This study aimed to develop a selective medium [referred to as *Actinomyces* and *Schaalia* Selective Medium (ASSM)] for the isolation of a broad range of *Actinomyces* and *Schaalia* species from samples mixed with resident bacteria. The composition of ASSM includes yeast extract, agar, brain heart infusion (BHI), levofloxacin (LVFX), fosfomycin (FOM), colistin (CL) and metronidazole (MNZ). Evaluation of the medium using 24 swab samples serially collected from the roots of the teeth of a healthy individual for whom metagenome sequencing data of a saliva sample are publicly available revealed that ASSM adjusted to concentrations of LVFX 0.5 mg l^−1^, FOM 5 mg l^−1^, CL 1 mg l^−1^ and MNZ 2 mg l^−1^ and cultured anaerobically at 35 °C for 7 days enabled the isolation of *Actinomyces* species from 37.5 % of the samples. The inclusion of CL and MNZ in ASSM can also be useful for samples harbouring other bacterial species. The selective isolation medium is expected to contribute to studies investigating the relationship between these bacteria and their pathogenesis or disease.

## Data Summary

All data associated with this work are reported within the article.

## Introduction

*Actinomyces* is one of the most diverse genera and is a part of the normal commensal flora in the mouth, gastrointestinal tract and female urogenital tract. *Actinomyces* is associated with diseases such as human actinomycosis [1] and medication-related osteonecrosis of the jaw [2,4]. In 2018, some species within the genus *Actinomyces* were reclassified as a new and distinct genus, *Schaalia*, through genomic analysis [5]. A selective medium for isolating *Actinomyces* and *Schaalia* should inhibit the growth of other bacteria.

To develop selective media for isolating *Actinomyces* species, commonly used basic media include those with added blood (e.g. Columbia agar with blood [6,8]) and those without blood [e.g. brain heart infusion (BHI) agar [7,9,11]). Zylber *et al*. [6], Ellen *et al*. [9] and Kornman *et al*. [10] developed selective media containing cadmium sulphate (CdSO_4_), sodium fluoride (NaF), colistin sulphate, nalidixic acid and metronidazole (MNZ) to isolate *Actinomyces viscosus* and *Actinomyces naeslundii* from dental plaques. However, CdSO_4_ is toxic and difficult to handle and dispose of. Beighton *et al*. [11] isolated *Actinomyces* from dental plaque using selective media containing colistin sulphate and NaF. Recently, Tsuzukibashi *et al*. [7] developed a selective medium containing ofloxacin (OFLX), fosfomycin (FOM), colistin (CL) and NaF to isolate *Actinomyces israelii*. However, NaF is also toxic. Except for Beighton’s selective medium, the media mentioned above were designed to isolate specific *Actinomyces* spp. Furthermore, the selective media developed by Ellen and Beighton may make isolation of these bacteria difficult because of their inability to inhibit the growth of Gram-positive cocci, such as *Staphylococcus* and *Enterococcus*.

The objective of this study was to develop a practical selective medium [referred to as *Actinomyces* and *Schaalia* Selective Medium (ASSM)] that can isolate various species of *Actinomyces* and *Schaalia* while inhibiting the growth of resident *Staphylococcus* and *Enterococcus* species.

## Methods

**Fig. 1.**
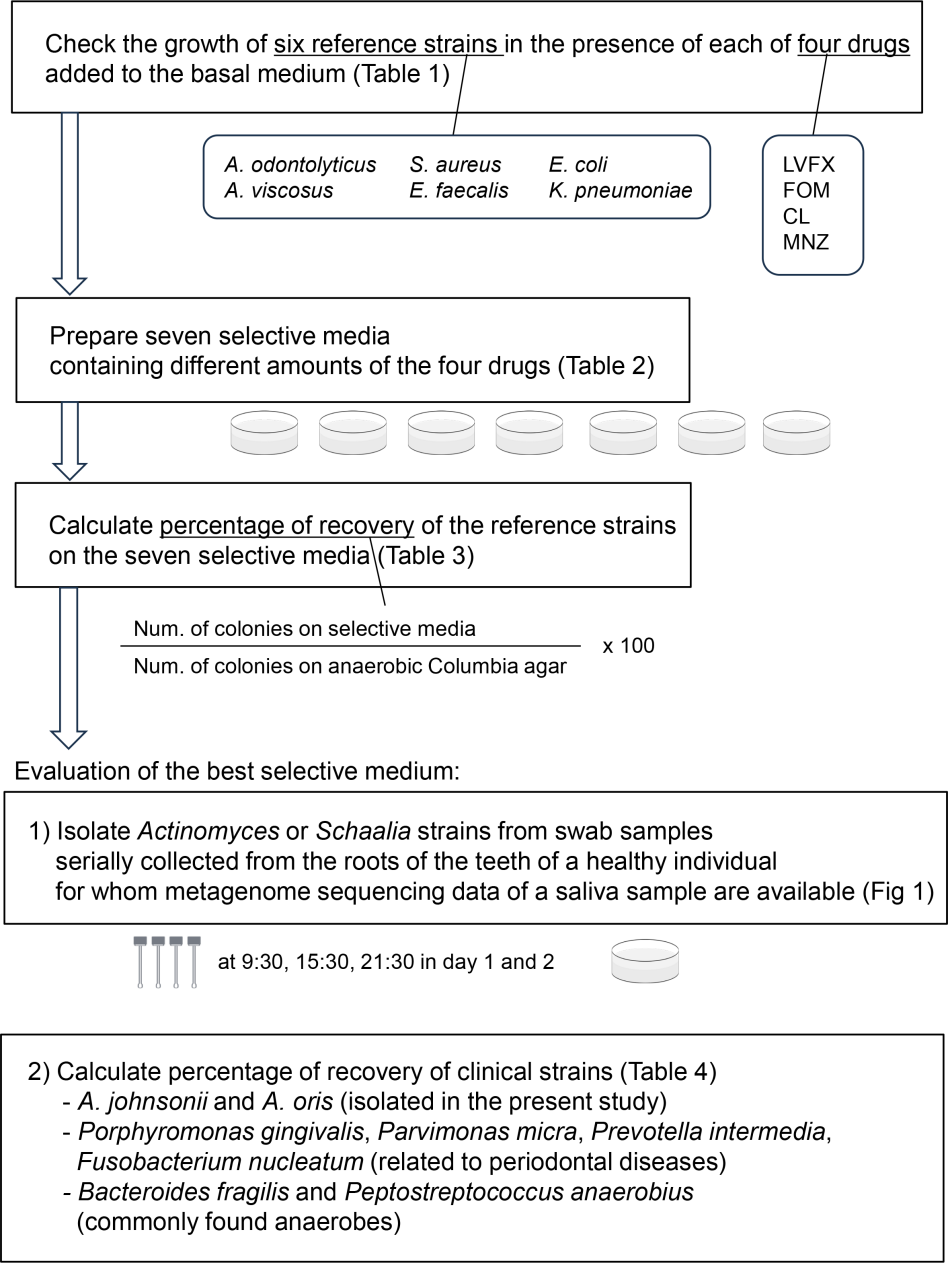
Flowchart summarizing the methods employed in the present study.

The following methods employed in the current study are outlined in a flowchart presented in Fig. 1.

### Reference strains

The following six reference strains were used to compare selective media explained below: *Actinomyces odontolyticus* (*Schaalia odontolytica*, according to the latest taxonomic classification) ATCC 17929, *A. viscosus* ATCC 15987, *Staphylococcus aureus* ATCC 29213, *Enterococcus faecalis* ATCC 29212, *Escherichia coli* ATCC 25922 and *Klebsiella pneumoniae* ATCC 700603. The reference strains were chosen from *Actinomyces* and *Schaalia* species, which are the focus of the present study, as well as common Gram-positive and Gram-negative species.

### Basal medium and antimicrobial drugs

The composition of the basal medium (YEBHI) was as follows: 1 litre of distilled water, 37 g BHI (BBL), 10 g yeast extract (Biokar) and 15 g agar (Nissui Pharmaceutical). The antimicrobial drugs added to the basal medium in the following experiments were levofloxacin (LVFX) from Tokyo Chemical Industry, FOM from Fujifilm Wako, CL from Sigma-Aldrich and MNZ from Fujifilm Wako.

### Evaluation of seven selective media using six reference strains

Preliminary tests were conducted to confirm growth of the following six reference strains in the presence of each of the four antimicrobial drugs. A dilution series of the four drugs (LVFX, FOM, CL and MNZ) was prepared and added to the basal medium of YEBHI. A total of 12 conditions were delineated (Table 1). For each condition, a bacterial liquid culture adjusted to McFarland 0.5 was spread onto the agar plate using an inoculation loop and incubated anaerobically at 35 °C for 7 days using an Anaeropack (Mitsubishi Gas Chemical). Growth on the media was evaluated using a four-tiered scale, (−) to (3+), based on the criteria outlined in Table 1. For each condition, we conducted two replicates and confirmed consistent results between them.

**Table 1.** Growth of the reference strains in the presence of levofloxacin, fosfomaicin, colistin and metronidazole

Ref. strain	YEBHI+LVFX			YEBHI+FOM			YEBHI+CL			YEBHI+MNZ			
	1	2	3	4	8	16	1	2	4	4	8	16	
	(mg l^–1^)			(mg l^–1^)			(mg l^–1^)			(mg l^–1^)			
*A. odontolyticus* (*Schaalia odontolytica*) ATCC 17929	3+	3+	3+	3+	3+	–	3+	3+	3+	3+	3+	3+	
*A. viscosus* ATCC 15987	3+	−	−	3+	3+	3+	3+	3+	3+	3+	3+	−	
*Staphylococcus aureus* ATCC 29213	−	−	−	3+	2+	2+	3+	3+	3+	3+	3+	3+	
*Enterococcus faecalis* ATCC 29212	−	−	−	3+	3+	3+	3+	3+	3+	3+	3+	3+	
*Escherichia coli* ATCC 25922	−	−	−	3+	3+	3+	−	−	−	3+	3+	3+	
*K. pneumoniae* ATCC 700603	3+	−	−	3+	3+	3+	−	−	−	3+	3+	3+	

The concentrations of each drug added to the basal medium (YEBHI) were as follows: ‘1 2 3’ (LVFX), ‘4 8 16’ (FOM), ‘1 2 4’ (CL), and ‘4 8 16’ (MNZ), presented in milligrams per literlitre (mg/L).

One loop of 0.5 McFarland bacterial concentration of the reference strains was spread onto the agar plates (corresponding to the columns in this table).

−: no growth; 1+: number of c.f.u. less than one-third of that on YEBHI; 2+: number of c.f.u. less than two-thirds of that on YEBHI; 3+: number of c.f.u. more than two-thirds of that on YEBHI.

For the main experiment with the reference strains, seven types of ASSMs containing different amounts of the four drugs were prepared based on preliminary test results (Table 2). For each of the six reference strains, a bacterial suspension diluted to a countable concentration using BHI for *Actinomyces* and pluronic surfactants for the other species (10 µl) was spread onto YEBHI and the seven types of ASSMs and then incubated anaerobically at 35 °C for 7 days using the Anaeropack. The percentage of recovery was calculated for each ASSM as follows: the number of colonies on ASSM divided by the number on anaerobic Columbia agar with RSB (Nippon Becton Dickinson) multiplied by 100. For each combination of reference strain and selective medium, we conducted replicates and used the average number calculated across the replicates as the denominator and numerator for calculating the percentage of recovery. For combinations where no colonies were observed on ASSM, we conducted two replicates. For the other combinations, we conducted at least three replicates, and increased the number of replicates when there was considerable variation in colony counts across replicates. Colony counts were determined using an Automatic Colony Counter Scan 4000 (Interscience).

**Table 2.** Composition of seven types of *Actinomyces* and *Schaalia* Selective Medium (ASSM) per litre

Ingredient	YEBHI	ASSM-1	ASSM-2	ASSM-3	ASSM-4	ASSM-5	ASSM-6	ASSM-7
Brain heart infusion (g)	37							
Yeast extract (g)	10							
Agar (g)	15							
Levofloxacin (LVFX) (mg)	0	1	1	1	1	0.5	0.5	0.5
Fosfomycin (FOM) (mg)	0	8	4	2	2	4	6	5
Colistin (CL) (mg)	0	1	1	1	0.5	1	1	1
Metronidazole (MNZ) (mg)	0	4	2	1	1	2	2	2

The same amounts of brain heart infusion, yeast extract, and agar were used in all seven types of medium.

### Sampling for isolation of *Actiomyces* and *Schaalia* strains

Swab samples from the roots of the teeth were collected from a healthy individual using a Puritan Opti-Swab LA116 (Puritan). Four samples were collected at 09 : 30, 15 : 30 and 21 : 30 h (representing morning, afternoon and night) on two consecutive days.

### Evaluation of the best selective medium

The swab samples were spread onto both the basal medium YEBHI and the selective medium ASSM with optimal drug composition and incubated anaerobically at 35 °C for 7 days using an Anaeropack. We then counted the number of samples from which the *Actinomyces* or *Schaalia* strains were isolated. The isolated bacteria were identified using matrix-assisted laser desorption/ionization time-of-flight (MALDI-TOF) MS using a MALDI Biotyper system (Bruker Daltonics).

The percentage of recovery was calculated for the selective medium ASSM with optimal drug composition using the following clinical strains: *Actinomyces johnsonii* and *Actinomyces oris* (isolated in the present study), as well as *Porphyromonas gingivalis*, *Parvimonas micra*, *Prevotella intermedia* and *Fusobacterium nucleatum* (all known to be related to periodontal diseases), and *Bacteroides fragilis* and *Peptostreptococcus anaerobius* (commonly found anaerobes). The clinical strains, excluding *A. jonsonii* and *A. oris*, were previously collected in 2016 as part of an unpublished project, where approximately 100 000 isolates across 89 species were collected from 1994 to 2016 by a pharmaceutical company and were subsequently transferred to the National Institute of Infectious Diseases.

The clinical strains showed the following MICs to LVFX: 1 µg ml^−1^ for *A. johnsonii*, 2 µg ml^−1^ for *A. oris*, 0.06 µg ml^−1^ for *Porphyromonas gingivalis*, 0.25 µg ml^−1^ for *Parvimonas micra*, 1 µg ml^−1^ for *Prevotella intermedia*, 1 µg ml^−1^ for *Fusobacterium nucleatum*, 1 µg ml^−1^ for *Bacteroides fragilis* and 0.25 µg ml^−1^ for *Peptostreptococcus anaerobius*.

## Results

The growth statuses of the six reference strains under each of the 12 conditions (three levels of dilution for LVFX, FOM, CL and MNZ added to the basal YEBHI medium) are shown in Table 1. *A. odontolyticus* (*Schaalia odontolytica*) ATCC 17929 showed an LVFX MIC >3 mg l^−1^. In contrast, growth inhibition of *A. viscosus* ATCC 15987 was observed at 2 mg l^−1^. FOM showed growth inhibition at 16 mg l^−1^ for *A. odontolyticus* (*Schaalia odontolytica*) ATCC 17929, whereas *A. viscosus* ATCC 15987 displayed growth even at a concentration of 16 mg l^−1^. Based on the results of this preliminary test, seven types of media were prepared and the main test was conducted using the reference strains. The results indicated that the medium ASSM-7 provided the optimal combination of recovery and inhibition rates (Table 3). Raw data of each replicate to calculate the percentage of recovery of the reference strains (Table 3) are shown in Table S1, available in the online version of this article.

**Table 3.** Percentage of recovery of reference strains on seven selective media

Ref.strain	Percentage of recovery on the following medium							
	YEBHI	ASSM-1	ASSM-2	ASSM-3	ASSM-4	ASSM-5	ASSM-6	ASSM-7
*A. odontolyticus* (*Schaalia odontolytica*) ATCC 17929	100	22	120	113	100	103	100	101
*A. viscosus* ATCC 15987	100	50	50	60	64	115	86	102
*Staphylococcus aureus* ATCC 29213	100	0	0	0	0	0	0	0
*Entercoccus faecalis* ATCC 29212	100	0	0	0	0	10	4	3
*Escherichia coli* ATCC 25922	100	0	0	0	0	0	0	0
*K. pneumoniae* ATCC 700603	100	0	0	0	0	0	0	0

**Fig. 2.**
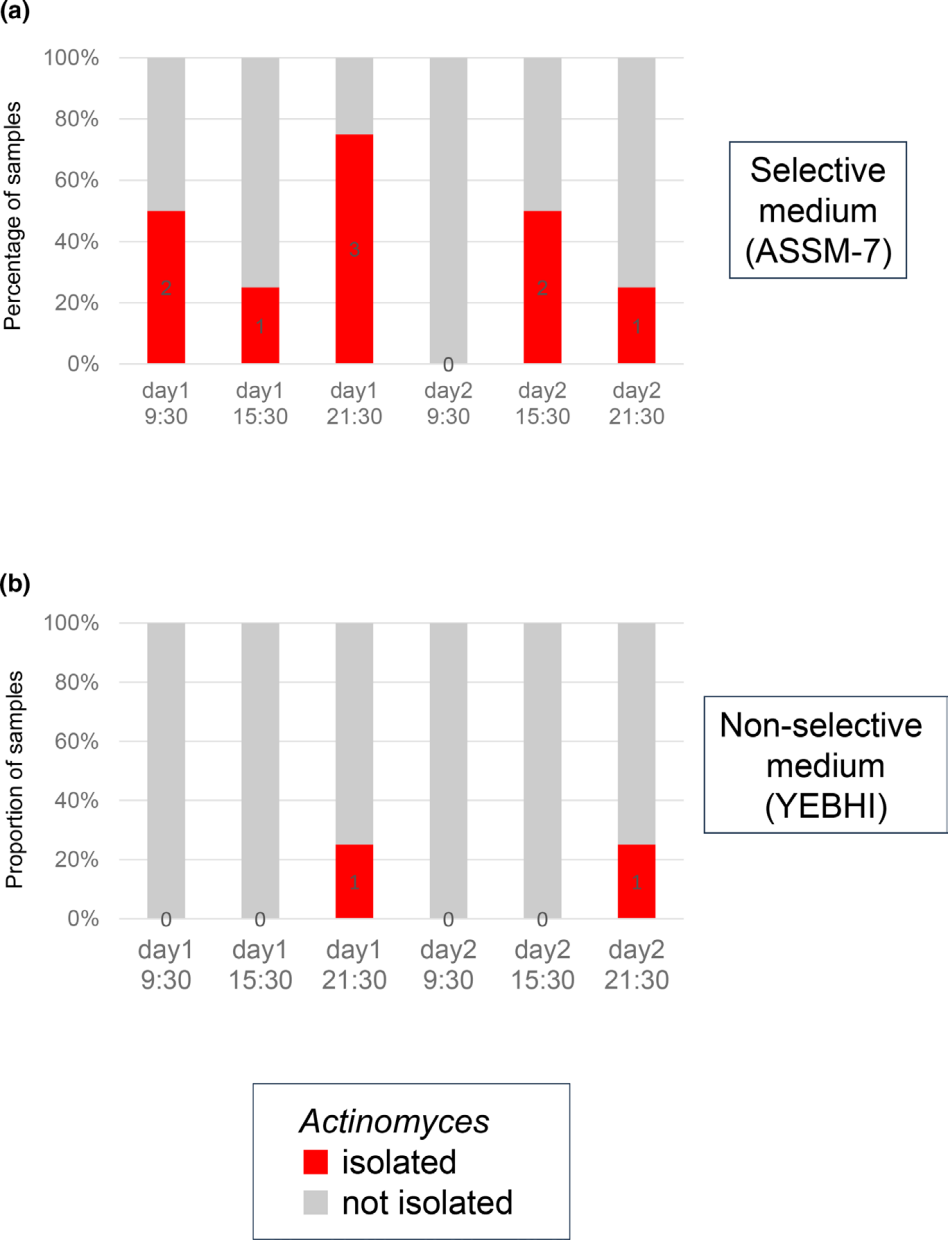
Comparison of the proportion of samples with *Actinomyces* isolation between the (**a**) selective and (**b**) non-selective media. Four oral swab samples collected from the roots of the teeth of a healthy individual at each time point (i.e. 09 : 30, 15 : 30 and 21 : 30 h on days 1 and 2) were tested, and those with *Actinomyces* isolation are indicated in red. The number of samples with *Actinomyces* isolation (from 0 to 3) at each time point is indicated in the bar chart in black.

Application of the selective medium ASSM-7 to oral swab samples serially collected from the roots of the teeth of a healthy individual revealed the isolation of *Actinomyces* species in 37.5 % (9/24) of the samples (Fig. 2). Specifically, *Actinomyces* species isolated using the selective medium ASSM-7 and identified through MALDI-TOF MS with the MALDI Biotyper system included *A. johnsonii* (*n*=7) and *A. oris* (*n*=2). This was substantially higher than the 8.3 % (2/24) of the non-selective YEBHI medium. The healthy individual participated in a previous study [12] that conducted metagenome sequencing of saliva samples. Examination of publicly available metagenome sequencing data (NCBI BioProject PRJDB9452, BioSample SAMD00202455) generated in the previous study showed a taxonomic abundance of *Actinomyces* at 5.8 %. The isolation of *Actinomyces* species using the selective medium ASSM-7 was possible irrespective of the sample collection time (i.e. 09 : 30, 15 :30 and 21 : 30 h).

The percentage of recovery using the selective medium ASSM-7 compared to the basal medium YEBHI is shown in Table 4 for clinical isolates, including *A. johnsonii* and *A. oris* isolated in the present study as well as for six oral bacterial species that are anaerobic or known to be related to periodontal diseases and were isolated and stored in a previous study. The percentage of recoveries of *A. johnsonii* and *A. oris* isolated in the present study ranged from 100 to 101 %. In contrast, the six other oral bacterial species that are anaerobic or known to be related to periodontal diseases did not grow on ASSM-7. Raw data of each replicate to calculate the percentage of recovery of the clinical strains (Table 4) are shown in Table S2.

**Table 4.** Percentage of recovery of clinical strains on selective medium ASSM-7 compared to anaero Columbia agar with RSB

Strain	Isolation		YEBHI	ASSM-7
		LVFX MIC (µg ml^–1^)	Percentage of recovery	
*Actinomyces johnsonii*	Present study	1	104	100
*Actinomyces oris*	Present study	2	100	101
*Parvimonas micra*	Previously	0.25	100	0
*Peptostreptococcus anaerobius*	Previously	0.25	0	0
*Prevotella intermedia*	Previously	0.5	0	0
*Porphyromonas gingivalis*	Previously	0.06	0	0
*Bacteroides fragilis*	Previously	1	100	0
*Fusobacterium nucleatum*	Previously	1	11	0

## Discussion

To develop selective media for isolating *Actinomyces* and *Schaalia* species from samples containing resident bacteria, including commensals, in the oral microbiota, we conducted an investigation using serially collected swab samples from the roots of the teeth of a healthy individual for whom metagenome sequencing data from a saliva sample are publicly available. As a result, a selective medium labelled ASSM-7 was devised by adding four drugs, LVFX, FOM, CL and MNZ, to the medium. By culturing these samples anaerobically at 35 °C for 7 days, *Actinomyces* species were isolated from 37.5 % of the samples using ASSM-7. We selected the healthy individual because they were the only individual for whom salivary metagenome sequencing data were publicly available and the serial sampling of oral swabs was feasible. Consequently, the difference in the proportion of samples with *Actinomyces* isolation between the non-selective medium and selective medium (ASSM-7) was not tested statistically. While this limitation is acknowledged in the present study, our ongoing follow-up study using the selective medium ASSM-7 has revealed that *Actinomyces* and *Schaalia* species were isolated from 13 out of 16 patients with medication-related osteonecrosis of the jaw mentioned in the Introduction.

To develop selective media for isolating *Actinomyces* species, commonly used basic media can be classified into those with added blood, such as Columbia agar with blood [6,8], and those without blood, such as BHI agar [7,9,11]. The basic medium used in the present study was YEBHI, prepared by adding yeast extract and agar to BHI. This medium offers a rapid alternative in cases where culture is required, because it does not use blood. Additionally, this medium is useful as a basic medium because it does not support the growth of certain species, including *Prevotella intermedia*, associated with periodontal disease (Table 4).

Anaerobic culture conditions were employed in this study for isolating these bacteria. Although many species of *Actinomyces* and *Schaalia* can grow under aerobic conditions, they may not exhibit typical branching filaments. Kornman *et al*. [10] reported that species such as *A. viscosus* and *A. naeslundii* can grow in CO_2_ culture but can also exhibit spherical forms on solid media. Although *A. oris* isolated in this study grew well under aerobic conditions, it did not show branching filaments. Thus, anaerobic culture is essential for observing typical morphological forms through Gram staining for help with identification. Moreover, anaerobic culture is effective in suppressing the growth of similar-looking species such as *Nocardia* and aerobic bacteria such as *Pseudomonas*.

Conventionally, selective media with additives such as CdSO_4_, NaF, CL, OFLX, FOM and MNZ have been used to isolate *Actinomyces* species [6,7, 9,11]. However, these selective media were designed for the isolation of specific *Actinomyces* species. In this study, four drugs – LVFX, FOM, CL and MNZ – were used to develop a medium for isolating a wide range of species. The rationale for this is as follows. As reported by Rama *et al*. [13], *Enterococcus faecalis* isolated from the subgingival gum areas showed resistance against MNZ at 4 µg ml^−1^, but was susceptible to ciprofloxacin, a fluoroquinolone, with a susceptibility rate of 89.4 % (42/47). Additionally, Beighton *et al*. [11] reported that a medium containing colistin sulphate and NaF did not inhibit the growth of *Staphylococcus* spp. In this study, for the main purpose of inhibiting the growth of these two species, LVFX, which is highly effective against *Entercoccus faecalis* [14]*,* and FOM, effective against *Staphylococcus aureus* and *Staphylococcus epidermidis* [15], were added. However, the addition of LVFX at 1 mg l^−1^ or FOM at 16 mg l^−1^ to YEBHI did not inhibit the growth of *K. pneumoniae* ATCC 700603, an extended-spectrum β-lactamase (ESBL)-producing bacterium. Therefore, CL was added to suppress such ESBL-producing bacteria. In addition, MNZ was added to inhibit the growth of anaerobic bacteria such as *B. fragilis*. MNZ is not effective in treating *Actinomyces* infections, as 94.3 % (347/368) of clinically isolated *Actinomyces* species are resistant [16,17]. Moreover, MNZ at 2.0 µg ml^−1^ was able to prevent the growth of *B. fragilis* group strains isolated from surgical sites, with a rate of 97.3 % (110/113) [18]. While caution is required when using multiple drugs in combination, owing to their potential antagonistic effects, a comparison of Table 1 (in the presence of a single drug) and Table 4 (in the presence of the four drugs used for the selective medium) suggests the absence of such effects in this study. In addition, although synergistic effects related to the four drugs have been reported, antagonistic effects have not been reported [19,20].

The ASSM-7 selective medium containing LVFX, FOM, CL and MNZ aims to allow isolation of multiple species with high sensitivity, thus compromising specificity. As a result, ASSM-7 selective medium was not able to completely suppress the growth of *Enterococcus faecalis* (Table 3). To isolate multiple species of *Actinomyces* and *Schaalia* while inhibiting the growth of these Gram-positive cocci, further development of a highly specific ASSM and its evaluation using clinical samples is warranted.

In this study, the utility of ASSM as a selective medium for *Actinomyces* and *Schaalia* species was investigated, and the optimal drug concentrations for inclusion were determined to be LVFX 0.5 mg l^−1^, FOM 5.0 mg l^−1^, CL 1.0 mg l^−1^ and MNZ 2.0 mg l^−1^. Through this investigation, it was confirmed that *Actinomyces* species could be isolated from swab samples from the roots of teeth including resident bacteria, demonstrating the effectiveness of the medium. Furthermore, owing to the addition of CL and MNZ, the medium is also useful for samples harbouring antimicrobial resistance (such as ESBL-producing bacteria), and other anaerobic bacteria. We expect that this medium will contribute to future studies investigating the relationship between these bacteria and their pathogenesis or disease.

## supplementary material

10.1099/acmi.0.000768.v3 Uncited Table S1.

## Abbreviations

ASSM: *Actinomyces* and *Schaalia* Selective Medium
BHI: brain heart infusion
CL: colistin
ESBL: extended-spectrum β-lactamase
FOM: fosfomycin
LVFX: levofloxacin
MALDI-TOF: matrix-assisted laser desorption/ionization time-of-flight
MNZ: metronidazole
